# High-intensity interval training as a cognitive enhancement strategy for executive function in overweight female university Students

**DOI:** 10.3389/fphys.2026.1886108

**Published:** 2026-09-16

**Authors:** Li Li, Lin Fang Sun

**Affiliations:** College of Sports and Art, Shandong Sport University, Shandong, Jinan, China

**Keywords:** cognitive flexibility, executive function, female university students, high-intensity interval training, overweight

## Abstract

**Background:**

Overweight female university students are vulnerable to sedentary behavior, reduced physical fitness, and impaired executive function. This study examined the effect of high-intensity interval training (HIIT) on executive function in overweight female university students.

**Methods:**

This randomized controlled pretest–posttest study involved 80 overweight female university students who were assigned to an exercise group (n = 40) or a control group (n = 40). The exercise group completed an eight-week supervised HIIT program, three sessions per week, while the control group maintained usual daily activities. Executive function was assessed using the Stroop Color–Word Test, Trail Making Test Part B, and Digit Span Test. Anthropometric variables were also measured.

**Results:**

Compared with the control group, the HIIT group demonstrated greater improvements in executive function. Stroop interference time decreased from 58.42 ± 7.35 to 49.86 ± 6.91 s in the HIIT group, compared with 57.89 ± 7.12 to 55.94 ± 7.28 s in the control group (group × time: *F* = 8.74, *p* = 0.004, ηp^2^ = 0.107). TMT-B completion time decreased from 74.35 ± 9.84 to 63.12 ± 8.76 s, compared with 73.91 ± 10.11 to 70.85 ± 9.94 s (*F* = 10.21, *p* = 0.002, ηp^2^ = 0.121). Digit Span total score increased from 11.24 ± 1.86 to 13.08 ± 1.94, compared with 11.31 ± 1.79 to 11.86 ± 1.83 (*F* = 7.69, *p* = 0.007, ηp^2^ = 0.095). Significant reductions in body weight, body mass index, and waist circumference were also observed in the HIIT group compared with the control group.

**Conclusion:**

High-intensity interval training is an effective and time-efficient strategy for improving executive function and anthropometric outcomes in overweight female university students.

## Introduction

Overweight is an increasing public health concern among university students, among whom prolonged sitting, irregular sleep, academic stress, unhealthy eating, and insufficient physical activity commonly coexist (Benaich et al., 2020; Castro et al., 2020). Globally, 2.5 billion adults were overweight in 2022, including more than 890 million living with obesity (Ezzati, 2024). Women show a slightly higher prevalence of overweight than men, making weight-related health risks particularly relevant during early adulthood (Pegington et al., 2023). Overweight female university students therefore represent an important population because metabolic, behavioral, and cognitive demands converge during a period when long-term lifestyle and self-regulatory behaviors are established (Telleria-aramburu and Arroyo-izaga, 2022).

Beyond metabolic and cardiovascular risk, excess body weight may also be associated with poorer executive function (Favieri et al., 2019), including inhibitory control, working memory, cognitive flexibility, attention regulation, planning, and goal-directed behavior (Diamond, 2014; Torre et al., 2024). These functions are important for learning, academic engagement, decision-making, and health-related self-regulation (Dohle et al., 2017; Manuhuwa et al., 2023). Executive dysfunction in individuals with overweight or obesity may involve low-grade inflammation, insulin resistance, impaired cerebrovascular regulation, and altered neurotrophic signaling (Nguyen et al., 2014). Reduced inhibitory control, working memory, and cognitive flexibility may also impair academic functioning and healthy behavioral regulation, suggesting a potentially bidirectional relationship between excess body weight and executive control (Hung, 2020; Yang et al., 2018).

Physical activity is a promising non-pharmacological strategy for improving executive function in individuals with overweight or obesity (Torre et al., 2024), although evidence remains heterogeneous across populations, exercise modalities, intensities, durations, and cognitive outcomes (Guo et al., 2024; Hidayat et al., 2025). High-intensity interval training (HIIT), characterized by repeated vigorous exercise interspersed with recovery, offers a time-efficient alternative to continuous exercise (Aljehani et al., 2022; Atakan et al., 2021; Gibala et al., 2012; Gibala and Jones, 2013). Its relatively short duration and minimal equipment requirements may be particularly suitable for university students facing academic and time constraints (Sian et al., 2021). Beyond physiological benefits, HIIT may also produce meaningful neurocognitive effects (Alves et al., 2021).

Evidence increasingly supports the cognitive benefits of HIIT. Acute and chronic HIIT have been associated with improvements in executive function across different populations (Ai et al., 2021; Hsieh et al., 2020), while a recent systematic review and meta-analysis reported favorable effects on cognitive performance, including executive function, memory, and information processing (Liu et al., 2024). Similar effects have been examined in children and adolescents (Liu et al., 2024; Reyes-amigo et al., 2022). However, existing evidence frequently involves heterogeneous populations and aggregated cognitive outcomes (Hsieh et al., 2020; Reyes-amigo et al., 2022). Overweight female university students remain comparatively underrepresented, and whether HIIT consistently improves specific executive-function domains in this population remains unclear. This question is particularly relevant because these students experience overlapping sedentary exposure, metabolic risk, and academic cognitive demands, while time-efficient exercise formats may be especially suitable for campus-based health promotion (Aljehani et al., 2022; Sian et al., 2021).

Therefore, this study investigated the effects of an eight-week supervised HIIT intervention on executive function in overweight female university students. Inhibitory control, selective attention, cognitive flexibility, and working memory were assessed as executive-function outcomes, while body weight, body mass index, and waist circumference were examined as secondary anthropometric outcomes. By combining a targeted population, domain-specific executive-function assessment, and a campus-relevant intervention, this study aimed to provide more specific evidence on the cognitive and physical-health effects of HIIT. We hypothesized that HIIT would produce greater improvements in executive function and greater reductions in anthropometric outcomes than usual daily activities (Guo et al., 2024; Liu et al., 2024).

### Study design

This study employed a two-arm, parallel-group randomized controlled pretest–posttest design to examine the effect of HIIT on executive function in overweight female university students. Participants were randomly allocated to either a HIIT group or a control group. The intervention was conducted over an eight-week period, with outcome assessments performed at baseline and immediately after the intervention. Executive function was defined as the primary outcome and was evaluated through measures of inhibitory control, cognitive flexibility, and working memory. Anthropometric variables were assessed as secondary outcomes to characterize changes in body-related health indicators following the intervention.

This design was selected because it allowed the comparison of changes over time between participants receiving the structured HIIT intervention and those maintaining their usual daily activities. The randomized controlled pretest–posttest approach was considered appropriate for determining whether HIIT produced measurable improvements in executive function beyond changes that might occur naturally over the study period. By focusing specifically on overweight female university students, the design directly addressed the research gap identified in the Introduction and enabled a targeted evaluation of HIIT as a potential cognitive enhancement strategy in a metabolically vulnerable and academically demanding population.

### Participants

A total of 80 overweight female undergraduate students were recruited from a university located in Jinan, Shandong Province, China, through campus advertisements, classroom announcements, and social media invitations. Eligible participants were women aged 18–24 years who were enrolled as active undergraduate students, had overweight status according to the Asian BMI classification, and had not participated in regular structured exercise during the previous three months. Overweight was defined as a BMI of 23.0–27.4 kg/m^2^, and eligibility was confirmed through direct measurement of body weight and height during screening. Participants were excluded if they reported cardiovascular disease, neurological disorders, musculoskeletal injuries, metabolic disease, psychiatric conditions, or other medical conditions that could affect exercise safety or cognitive assessment. They were also excluded if they were taking medications that could influence cognitive performance, heart-rate response, metabolism, or exercise tolerance, or if they were currently participating in another structured exercise or weight-management program.

Screening included demographic assessment, anthropometric measurement, health-history evaluation, medication-use assessment, and confirmation of physical activity status during the previous three months. Participants who fulfilled the eligibility criteria and provided written informed consent were enrolled in the study. Following baseline assessment, participants were randomly assigned to either the HIIT group or the control group, with 40 participants allocated to each group. The sample size was estimated using G*Power version 3.1 for a repeated-measures analysis of variance with two groups and two time points. Assuming a medium effect size, an alpha level of 0.05, and statistical power of 0.80, the required sample size was considered adequate to detect a group × time interaction. To reduce the risk of insufficient power due to potential dropout or incomplete data, 80 participants were recruited and randomized.

### Randomization procedure

Following completion of baseline assessments, eligible participants were randomly allocated to either the HIIT group or the control group in a 1:1 allocation ratio. The randomization sequence was generated using a computer-based random number generator by an independent researcher who was not involved in participant recruitment, intervention delivery, outcome assessment, or data analysis. To maintain balance between groups, block randomization was applied. Allocation concealment was ensured using sequentially numbered, opaque, sealed envelopes prepared by the independent researcher. Each envelope contained the group assignment and was opened only after the participant had completed all baseline assessments and had been confirmed as eligible for inclusion. This procedure was used to minimize selection bias and prevent prediction of group assignment during recruitment. Due to the nature of the exercise intervention, blinding of participants and exercise instructors was not feasible. However, outcome assessors responsible for administering executive function tests and researchers involved in data analysis were blinded to group allocation. Participants were also instructed not to disclose their group assignment during post-intervention assessments. These procedures were implemented to reduce detection bias and strengthen the internal validity of the trial.

### High-intensity interval training intervention

Participants assigned to the intervention group completed a supervised HIIT program three times per week for eight consecutive weeks, resulting in a total of 24 planned sessions (Burgomaster et al., 2008; Gibala et al., 2012; Gibala and Jones, 2013). The eight-week intervention period was selected to balance physiological relevance and practical feasibility (Gibala et al., 2012; MacInnis and Gibala, 2017). A shorter duration, such as four weeks, may be insufficient to produce stable training-related changes in executive function and anthropometric outcomes, whereas a longer duration, such as twelve weeks, may increase participant burden and dropout risk among university students with academic commitments. Therefore, an eight-week program was considered appropriate to provide repeated exposure to HIIT across 24 supervised sessions while remaining feasible within a campus-based intervention context (Milanovic´ et al., 2015; Yamaner et al., 2025). All sessions were conducted in a university exercise facility and supervised by trained exercise instructors to ensure correct technique, adherence to the protocol, and participant safety. Before the intervention began, participants completed a familiarization session to learn the exercise movements, practice the interval structure, and receive instructions regarding perceived exertion and safety procedures.

Each training session lasted approximately 30 minutes and consisted of a 5-minute warm-up, 20 minutes of HIIT, and a 5-minute cool-down (Gibala et al., 2012; Little et al., 2014). The warm-up included light aerobic movements and dynamic stretching. The main interval protocol consisted of 10 cycles of 1-minute high-intensity exercise performed at 85–95% of maximal heart rate, followed by 1-minute active recovery at 50–60% of maximal heart rate. The exercise activities included bodyweight-based aerobic and functional movements, such as jumping jacks, high knees, mountain climbers, squat jumps, and modified burpees (Jr et al., 2017; Müller et al., 2017). Movements were selected because they required minimal equipment, were feasible in a campus-based setting, and involved large muscle groups to elicit sufficient cardiovascular demand.

Exercise intensity was monitored continuously using wearable heart-rate monitors. Maximal heart rate was estimated using the age-predicted formula, and participants were instructed to maintain the prescribed target heart-rate zone during each interval (Bacon et al., 2013; Garber et al., 2011). Rating of perceived exertion was also recorded during sessions as a complementary measure of internal exercise load (Gerage et al., 2012; She et al., 2015). HIIT were targeted at a perceived exertion corresponding to hard-to-very-hard effort, while recovery intervals were maintained at light-to-moderate effort. When participants were unable to maintain proper technique or target intensity, exercise movements were modified while preserving the intended cardiovascular intensity (Atakan et al., 2021; MacInnis and Gibala, 2017). Safety was monitored throughout the intervention. Before each session, participants were asked about fatigue, musculoskeletal discomfort, illness, or other symptoms that could affect exercise participation. Sessions were stopped or modified if participants reported dizziness, chest discomfort, excessive shortness of breath, abnormal pain, or any adverse symptoms (Hollerbach et al., 2021; Laursen and Jenkins, 2002). Attendance, exercise completion, heart-rate response, perceived exertion, and adverse events were recorded at every session.

Participants in the control group were instructed to maintain their usual daily activities and avoid initiating any new structured exercise or weight-management program during the eight-week study period. To monitor compliance, control participants were asked to report any changes in physical activity habits during the intervention. Exercise adherence in the intervention group was calculated as the percentage of completed sessions out of the 24 planned sessions. Participants who completed at least 85% of the scheduled sessions were considered adherent for per-protocol analysis.

### Executive function assessment

Executive function was the primary outcome and was assessed at baseline and after the eight-week intervention using standardized neuropsychological tests administered in a quiet laboratory under standardized environmental conditions (Diamond, 2014; Faria et al., 2015). The assessment battery comprised the Stroop Color–Word Test, Trail Making Test Part B (TMT-B), and Digit Span Test, which collectively evaluated inhibitory control, selective attention, cognitive flexibility, task switching, and working memory (Periáñez et al., 2009; Scarpina and Tagini, 2017; Woods et al., 2012). All assessments were administered individually by trained assessors who were blinded to group allocation.

The Stroop Color–Word Test was used to assess inhibitory control and selective attention (Macleod, 1991; Scarpina and Tagini, 2017; Stroop, 1992). A computerized Stroop task was administered using four color-word stimuli: RED, BLUE, GREEN, and YELLOW. Stimuli were presented in either congruent or incongruent ink colors on a computer screen. Participants first completed a practice block of eight trials to ensure task comprehension, followed by two experimental blocks consisting of 40 congruent and 40 incongruent stimuli presented in randomized order. Participants were instructed to identify the ink color as quickly and accurately as possible while ignoring the semantic meaning of the printed word (Parris et al., 2022; Scarpina and Tagini, 2017). Each trial began with the presentation of a fixation cross, followed by the color-word stimulus, which remained on the screen until the participant responded or until the maximum response window elapsed. Completion time and interference performance were recorded, with lower interference time indicating superior inhibitory control (Parris, 2014; Scarpina and Tagini, 2017). Incorrect responses were immediately corrected, and testing continued without restarting the task.

Cognitive flexibility and task-switching ability were assessed using the standard paper-and-pencil version of the Trail Making Test Part B (Gündüz et al., 2021; Kortte et al., 2002). The test consisted of 25 targets (numbers 1–13 and letters A–L) distributed randomly across a single sheet. Participants were instructed to connect the circles sequentially by alternating between numbers and letters (1–A–2–B–3–C…) as rapidly and accurately as possible without lifting the pencil from the paper (Bowie and Harvey, 2006; Tombaugh, 2004). Before the recorded trial, participants completed a brief practice task containing eight targets to familiarize themselves with the alternating sequence. Completion time (s) was recorded as the primary outcome, with shorter completion time representing better cognitive flexibility (Gündüz et al., 2021; Periáñez et al., 2009). Errors were immediately indicated by the assessor, and participants were instructed to continue from the last correct connection.

Working memory was evaluated using the Digit Span Test (Hester et al., 2012; Woods et al., 2012). Digit sequences ranging from three to nine digits were presented orally by the assessor at a standardized rate of approximately one digit per second. The assessment included both Digit Span Forward and Digit Span Backward conditions, with two trials administered at each sequence length (Conway et al., 2005; Egeland, 2015). Testing was discontinued after failure on both trials of the same sequence length. The total score represented the sum of correctly recalled sequences across both conditions, with higher scores indicating greater working memory capacity (Egeland, 2015; Hester et al., 2012).

Executive function assessments were administered in a fixed sequence consisting of the Stroop Color–Word Test, Trail Making Test Part B, and Digit Span Test. This order was maintained across all participants and both assessment sessions to ensure procedural consistency. Although task order was not counterbalanced, standardized instructions, identical testing conditions, assessor blinding, and two-minute seated rest intervals between tasks were implemented to minimize procedural variability, mental fatigue, and immediate carryover effects.

Participants were instructed to refrain from vigorous physical activity for 24 hours, caffeine consumption for at least 12 hours, and alcohol consumption for 24 hours before testing. They were also instructed to obtain at least seven hours of sleep on the night preceding each assessment. Post-intervention testing was conducted at least 24 hours after the final exercise session to minimize acute exercise effects and better reflect chronic training adaptations. All assessments were conducted at approximately the same time of day for each participant to reduce potential circadian influences on cognitive performance.

### Anthropometric measurements

Anthropometric measurements were obtained at baseline and after the eight-week intervention as secondary physical outcomes. Body weight was measured to the nearest 0.1 kg using a calibrated digital scale (Tanita HD-661, Tanita Corporation, Tokyo, Japan), and height was measured to the nearest 0.1 cm using a portable stadiometer (Seca 213, Seca GmbH & Co. KG, Hamburg, Germany). Participants were assessed while wearing light clothing and no shoes. Body mass index was calculated as body weight in kilograms divided by height in meters squared, and overweight status was classified according to the BMI criteria used for participant eligibility (Nishida, 2004).

Waist circumference was measured using a non-elastic measuring tape (Seca 201, Seca GmbH & Co. KG, Hamburg, Germany) at the midpoint between the lowest rib and the iliac crest. Measurements were taken with participants standing upright, feet shoulder-width apart, and at the end of a normal expiration (Nishida et al., 2010; Singh et al., 2022). The tape was positioned horizontally around the abdomen without compressing the skin (Pettitt et al., 2013). Each measurement was performed twice, and the average value was used for analysis (Ashwell et al., 2011). If the difference between repeated waist circumference measurements exceeded 0.5 cm, a third measurement was taken and the closest two values were averaged. All assessments were conducted by trained assessors using standardized procedures to minimize measurement error. All equipment was checked before each assessment session and calibrated according to the manufacturers’ instructions where applicable.

### Exercise intensity monitoring

Exercise intensity was monitored during each intervention session using wearable heart-rate monitors. Maximal heart rate was estimated using the age-predicted formula HRmax = 220 − age. Because age-predicted HRmax equations may not fully account for interindividual variability in heart-rate responses, the estimated HRmax was used as a practical reference for prescribing and monitoring exercise intensity rather than as an individualized measure of true maximal heart rate. Participants were instructed to perform the high-intensity intervals at 85–95% of HRmax and the active recovery periods at 50–60% of HRmax (Bouaziz et al., 2020; Liu et al., 2024). Heart-rate responses were recorded during each session to verify adherence to the prescribed intensity zones and to support intervention fidelity. To complement heart-rate monitoring and account for individual variation in physiological responses to exercise, rating of perceived exertion was assessed using the Borg 6–20 scale as an additional measure of internal exercise load (She et al., 2015). High-intensity intervals were targeted at hard-to-very-hard effort, whereas recovery intervals were maintained at light-to-moderate effort (Jr et al., 2017; Milanovic´ et al., 2015). Accordingly, exercise intensity was evaluated using both heart-rate responses and perceived exertion rather than relying exclusively on the age-predicted HRmax. If participants failed to reach the target intensity, instructors adjusted movement tempo or provided standardized encouragement while ensuring proper exercise technique. If participants reported excessive fatigue, dizziness, chest discomfort, or other adverse symptoms, exercise intensity was reduced or the session was discontinued. Heart rate, perceived exertion, session completion, and adverse events were documented throughout the intervention.

### Statistical analysis

Statistical analyses were performed using SPSS version 26.0. Continuous variables were presented as mean ± standard deviation, while categorical variables were reported as frequency and percentage. Executive function outcomes, including Stroop Color–Word Test performance, Trail Making Test Part B completion time, and Digit Span score, were treated as primary outcomes. Anthropometric variables, including body weight, body mass index, and waist circumference, were analyzed as secondary outcomes. Normality was assessed using the Shapiro–Wilk test and visual inspection of Q–Q plots. Homogeneity of variance was examined using Levene’s test. Baseline differences between groups were analyzed using independent sample t-tests for continuous variables and chi-square tests for categorical variables, where appropriate. Standardized mean differences were also reported for baseline variables to describe the magnitude of between-group imbalance, with smaller values indicating better baseline comparability.

The effects of the intervention were examined using two-way repeated-measures ANOVA, with group as the between-subject factor and time as the within-subject factor. The group × time interaction was considered the primary indicator of intervention effectiveness. When significant interaction effects were observed, Bonferroni-adjusted *post hoc* comparisons were conducted. Effect sizes were reported as partial eta squared (ηp^2^), and mean differences with 95% confidence intervals were presented where appropriate. Statistical significance was set at p < 0.05. The primary analysis included participants with complete baseline and post-intervention data, while adherence and dropout were reported descriptively to support interpretation of intervention fidelity.

### Ethical considerations

This study was conducted in accordance with the ethical principles of the Declaration of Helsinki. The study protocol was reviewed and approved by the Ethics Committee of Shandong Sport University prior to participant recruitment and data collection. All participants received detailed information about the study objectives, procedures, potential benefits, possible risks, and their rights as research participants. Written informed consent was obtained before enrollment. Participation was voluntary, and participants were informed that they could withdraw from the study at any time without any consequences. Given the high-intensity nature of the exercise intervention, participant safety was monitored carefully. Eligibility screening was conducted before participation, and all exercise sessions were supervised by trained personnel. Heart rate, perceived exertion, fatigue, discomfort, and adverse events were monitored during each session. Exercise intensity was reduced or discontinued if participants reported dizziness, chest discomfort, excessive shortness of breath, abnormal pain, or other adverse symptoms. Participant confidentiality was maintained by coding and securely storing all research data.

## Results

### Participant flow and exercise adherence

The participant flow is summarized in **Figure 1**. Briefly, 80 eligible female university students were randomized equally to the HIIT and control groups. Participant retention was high across the eight-week study period, with only a small number of participants not completing post-intervention assessment. The final analysis included 75 participants, consisting of 38 participants in the exercise group and 37 participants in the control group. Exercise adherence was good, with the intervention group attending an average of 91.6% of the 24 scheduled sessions. No serious adverse events were reported, indicating that the supervised intervention was well tolerated.

**Figure 1:**
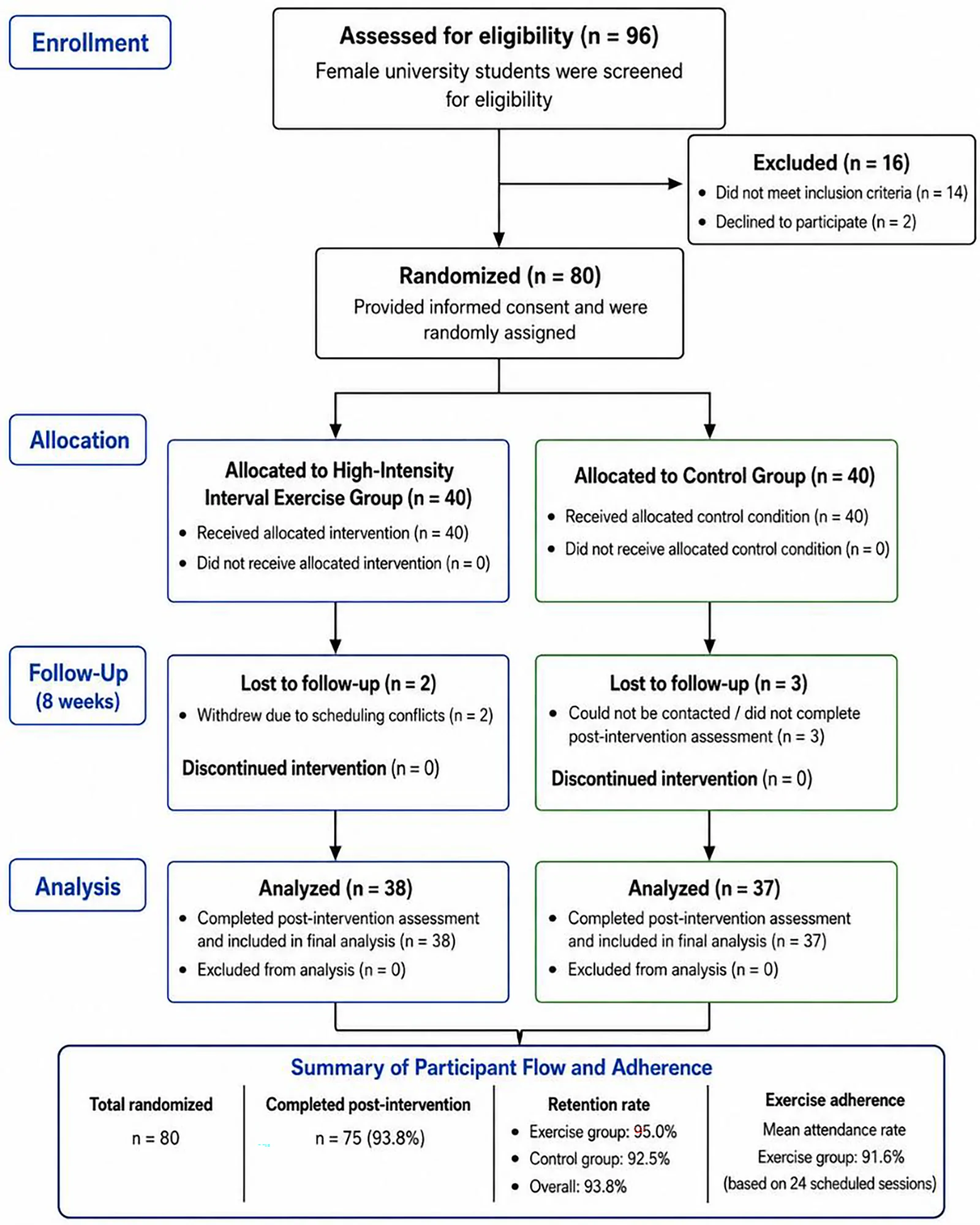
CONSORT flow diagram of participant recruitment, randomization, follow-up, and final analysis in the HIIT intervention study.

### Baseline characteristics

Baseline characteristics of the randomized participants are summarized in **Table 1**. Continuous variables are presented as mean ± standard deviation, while categorical variables are presented as n (%). Baseline demographic, anthropometric, executive function, and categorical participant characteristics were generally similar between the HIIT and control groups. No significant baseline differences were observed between groups for continuous or categorical variables. Standardized mean differences were small, indicating no substantial baseline imbalance across age, body composition indicators, executive function outcomes, year of study, academic major, or residence status.

**Table 1:** Baseline characteristics of participants.

Variable	Exercise group (n = 40)	Control group (n = 40)	P-value	SMD
Continuous variables				
Age (years)	20.48 ± 1.62	20.31 ± 1.57	0.641	0.11
Body weight (kg)	65.84 ± 5.72	66.11 ± 5.94	0.832	0.05
Height (m)	1.60 ± 0.05	1.61 ± 0.06	0.517	0.18
BMI (kg/m^2^)	25.71 ± 1.24	25.58 ± 1.31	0.673	0.10
Waist circumference (cm)	82.46 ± 5.18	82.91 ± 5.41	0.714	0.08
Stroop interference time (s)	58.42 ± 7.35	57.89 ± 7.12	0.748	0.07
TMT-B completion time (s)	74.35 ± 9.84	73.91 ± 10.11	0.846	0.04
Digit Span total score	11.24 ± 1.86	11.31 ± 1.79	0.862	0.04
Categorical variables				
Year of study, n (%)			0.812	—
Year 1	9 (22.5)	10 (25.0)		
Year 2	11 (27.5)	12 (30.0)		
Year 3	12 (30.0)	11 (27.5)		
Year 4	8 (20.0)	7 (17.5)		
Academic major, n (%)			0.764	—
Health-related fields	14 (35.0)	13 (32.5)		
Non-health fields	26 (65.0)	27 (67.5)		
Residence status, n (%)			0.691	—
On-campus	18 (45.0)	19 (47.5)		
Off-campus	22 (55.0)	21 (52.5)		

Continuous variables are presented as mean ± standard deviation, and categorical variables are presented as n (%). BMI = body mass index; TMT-B = Trail Making Test Part B; SMD = standardized mean difference. Independent-samples t-tests were used for continuous variables, and chi-square tests were used for categorical variables. No significant baseline differences were observed between groups.

### Executive function outcomes

Executive function outcomes are summarized in **Table 2** and illustrated in **Figure 2**. For Stroop interference time and Trail Making Test Part B completion time, lower values indicate better performance, whereas higher Digit Span scores indicate better working memory capacity. Significant group × time interactions were observed for all executive function outcomes. Stroop interference time showed a significant interaction effect (*F* = 8.74, *p* = 0.004, ηp^2^ = 0.107), with a greater reduction in the exercise group than in the control group. Trail Making Test Part B completion time also demonstrated a significant group × time interaction (*F* = 10.21, *p* = 0.002, ηp^2^ = 0.121), with a greater decrease in completion time in the exercise group than in the control group. Similarly, Digit Span total score showed a significant interaction effect (*F* = 7.69, *p* = 0.007, ηp^2^ = 0.095), with a larger increase observed in the exercise group. Bonferroni-adjusted *post hoc* analyses showed significant decreases in Stroop interference time and Trail Making Test Part B completion time and a significant increase in Digit Span total score in the exercise group (all *p* < 0.001), whereas changes in the control group were not statistically significant.

**Table 2:** Changes in executive function outcomes following the intervention.

Variable	Group	Pre-intervention	Post-intervention	Mean change	Within-group p-value	Group × Time interaction
Stroop interference time (s)	Exercise	58.42 ± 7.35	49.86 ± 6.91	-8.56	<0.001	F = 8.74; p = 0.004; ηp^2^ = 0.107
	Control	57.89 ± 7.12	55.94 ± 7.28	-1.95	0.182	
Trail Making Test Part B completion time (s)	Exercise	74.35 ± 9.84	63.12 ± 8.76	-11.23	<0.001	F = 10.21; p = 0.002; ηp^2^ = 0.121
	Control	73.91 ± 10.11	70.85 ± 9.94	-3.06	0.096	
Digit Span total score	Exercise	11.24 ± 1.86	13.08 ± 1.94	1.84	<0.001	F = 7.69; p = 0.007; ηp^2^ = 0.095
	Control	11.31 ± 1.79	11.86 ± 1.83	0.55	0.141	

**Figure 2:**
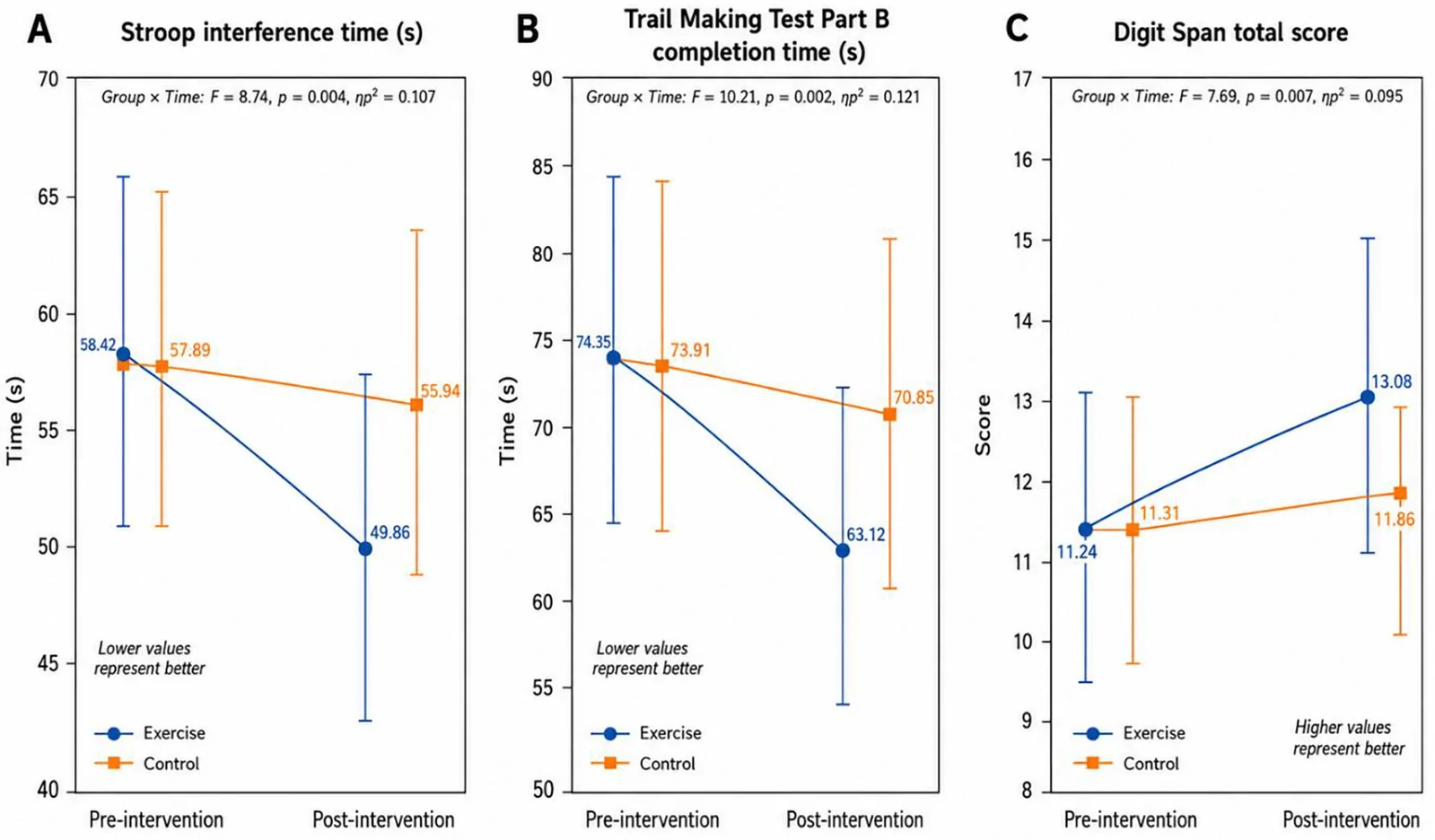
Changes in Executive Function Outcomes Before and After the Eight-Week HIIT Intervention. **(A)** Stroop interference time; **(B)** Trail Making Test Part B (TMT-B) completion time; and (C) Digit Span total score. Values are presented as mean ± SD. Lower values indicate better performance for the Stroop interference test and TMT-B, whereas higher values indicate better performance for the Digit Span test. HIIT = high-intensity interval training; TMT-B = Trail Making Test Part B; ηp^2^ = partial eta squared.

### Anthropometric outcomes

Anthropometric outcomes are presented in **Table 3**. Significant group × time interactions were found for body weight (*F* = 6.82, *p* = 0.011, ηp^2^ = 0.083), BMI (*F* = 6.57, *p* = 0.012, ηp^2^ = 0.081), and waist circumference (*F* = 9.14, *p* = 0.003, ηp^2^ = 0.111). The exercise group showed greater decreases in body weight, BMI, and waist circumference than the control group. Bonferroni-adjusted *post hoc* analyses showed significant decreases in all three anthropometric outcomes in the exercise group (all *p* < 0.001), whereas changes in the control group were not statistically significant.

**Table 3:** Changes in anthropometric outcomes following the intervention.

Variable	Group	Pre-intervention	Post-intervention	Mean change	Within-group p-value	Group × Time interaction
Body weight (kg)	Exercise	65.84 ± 5.72	63.91 ± 5.48	-1.93	<0.001	F = 6.82; p = 0.011; ηp^2^ = 0.083
	Control	66.11 ± 5.94	65.82 ± 5.87	-0.29	0.284	
BMI (kg/m^2^)	Exercise	25.71 ± 1.24	24.96 ± 1.19	-0.75	<0.001	F = 6.57; p = 0.012; ηp^2^ = 0.081
	Control	25.58 ± 1.31	25.49 ± 1.29	-0.09	0.317	
Waist circumference (cm)	Exercise	82.46 ± 5.18	78.94 ± 4.97	-3.52	<0.001	F = 9.14; p = 0.003; ηp^2^ = 0.111
	Control	82.91 ± 5.41	82.37 ± 5.29	-0.54	0.228	

## Discussion

This study examined the effects of an eight-week supervised HIIT intervention on executive function in overweight female university students. The main findings showed greater gains in inhibitory control, selective attention, cognitive flexibility, and working memory in the exercise group than in the control group, accompanied by significant reductions in body weight, BMI, and waist circumference. These findings support growing evidence that HIIT may positively influence executive function while providing additional physical health benefits in populations with overweight status (Ai et al., 2021; Hsieh et al., 2020; Liu et al., 2024).

The reductions in Stroop interference time and Trail Making Test Part B completion time indicate better performance in inhibitory control, selective attention, cognitive flexibility, and task switching. These findings are consistent with previous evidence showing beneficial effects of HIIT on executive-function outcomes across different populations (Kang et al., 2022; Liu et al., 2024). Improved performance on these tasks may reflect greater efficiency of executive control processes relevant to attention regulation and adaptation to changing cognitive demands (Lee et al., 2019; Liu et al., 2024). However, because only performance-based cognitive measures were used, the present findings demonstrate changes in executive-function task performance rather than direct changes in underlying neural processes.

Working memory, assessed using the Digit Span Test, also increased following the intervention. This finding is consistent with broader evidence indicating that exercise may benefit working-memory and other executive-function domains in individuals with overweight or obesity (Castell-Alcalá et al., 2022; Guo et al., 2024). Previous systematic reviews have similarly suggested positive effects of physical activity on executive function, although substantial heterogeneity remains across exercise protocols, populations, and cognitive measures (Liu et al., 2022; Torre et al., 2024; Xu et al., 2023). The present findings extend this evidence by demonstrating domain-specific executive-function responses to a time-efficient HIIT program in overweight female university students.

Several physiological and neurobiological pathways may plausibly contribute to these cognitive effects. Intermittent vigorous exercise may enhance cerebral perfusion, oxygen delivery, neurotransmitter activity, and neuroplastic processes associated with executive control (Ai et al., 2021; Basso and Suzuki, 2017; Smith and Ainslie, 2017). High-intensity exercise has also been associated with increased circulating brain-derived neurotrophic factor, which contributes to synaptic plasticity and cognitive function (Lu et al., 2014; Miranda et al., 2019; Szuhany et al., 2015). Improvements in executive-function task performance observed in the present study may therefore reflect adaptations within these interconnected systems. However, cerebral blood flow, neuroimaging, electrophysiological activity, BDNF, inflammatory markers, insulin sensitivity, and catecholamines were not measured; therefore, these mechanisms should be regarded as biologically plausible explanations rather than mechanisms directly demonstrated by this study.

The intervention also produced modest but significant reductions in body weight, BMI, and waist circumference (Bellicha et al., 2021; Thorogood et al., 2011). The relatively modest reduction in body weight is consistent with the short intervention period and absence of structured dietary restriction, whereas the greater reduction in waist circumference suggests that central anthropometric measures may respond more readily to this type of intervention (Thorogood et al., 2011). Because body composition, dietary intake, and metabolic biomarkers were not assessed, these anthropometric changes cannot be interpreted as evidence of changes in fat mass, insulin sensitivity, or inflammatory status. Nevertheless, the approximately 30-minute supervised sessions performed three times weekly highlight the practical potential of HIIT as a time-efficient approach for supporting cognitive and physical health in university settings.

Several strengths and limitations should be considered. The randomized controlled pretest–posttest design, supervised sessions, heart-rate monitoring, and assessment of multiple executive-function domains strengthened intervention fidelity and internal validity. However, the eight-week duration limits conclusions regarding long-term effects, and the absence of neurophysiological and metabolic biomarkers restricts mechanistic interpretation. Dietary intake, sleep quality, menstrual cycle phase, and psychological stress were not strictly controlled, and the fixed order of cognitive assessments may have introduced practice, fatigue, or carryover effects. In addition, inclusion of only overweight female university students limits generalizability to males, individuals with obesity, and other age groups. These considerations are particularly relevant given the methodological heterogeneity reported across previous exercise–cognition studies (Hsieh et al., 2020; Torre et al., 2024).

Future studies should incorporate longer follow-up periods, larger and more diverse samples, and neuroimaging, electrophysiological, vascular, and circulating biomarker assessments to clarify the mechanisms underlying HIIT-related cognitive responses. Future trials should also examine whether changes in body composition, fitness, inflammation, or insulin sensitivity mediate executive-function outcomes and should counterbalance cognitive-task order to minimize systematic testing effects (Liu et al., 2024; Torre et al., 2024; Xu et al., 2023). Overall, the present findings indicate that eight weeks of supervised HIIT was associated with better executive-function task performance and modest reductions in anthropometric measures among overweight female university students, supporting its potential as a practical and time-efficient campus-based exercise strategy.

## Conclusion

An eight-week supervised HIIT intervention produced greater improvements in executive function among overweight female university students compared with usual daily activities. Improvements were observed in inhibitory control, selective attention, cognitive flexibility, and working memory, while modest favorable changes in body weight, BMI, and waist circumference were also found as secondary outcomes. These findings suggest that HIIT may be a practical and time-efficient strategy for supporting cognitive function in overweight young women within university settings. Further studies with longer follow-up periods and neurophysiological or metabolic measurements are needed to confirm the sustainability and mechanisms of these effects.

## Data Availability

The original contributions presented in the study are included in the article/supplementary material. Further inquiries can be directed to the corresponding author.
